# Rationale and design of South Asian Birth Cohort (START): a Canada-India collaborative study

**DOI:** 10.1186/1471-2458-13-79

**Published:** 2013-01-28

**Authors:** Sonia S Anand, Anil Vasudevan, Milan Gupta, Katherine Morrison, Anura Kurpad, Koon K Teo, Krishnamachari Srinivasan

**Affiliations:** 1 McMaster University, 1280 Main St West MDCL 3200, L8S 4K1, Hamilton, ON, Canada; 2 Population Genomics Program, Chanchlani Research Centre, McMaster University, Hamilton, Canada; 3 Population Health Research Institute, Hamilton Health Sciences and McMaster University, Hamilton, Canada; 4 Canadian Cardiovascular Research Network, Brampton, Ontario; 5 St John’s Research Institute and St. John’s Medical College, Bangalore, India

**Keywords:** Birth cohort, South Asian, Adiposity, Insulin resistance, Early origins, India, Canada

## Abstract

**Background:**

People who originate from the Indian subcontinent (South Asians) suffer among the highest rates of type 2 diabetes in the world. Prior evidence suggests that metabolic risk factors develop early in life and are influenced by maternal and paternal behaviors, the intrauterine environment, and genetic factors. The South Asian Birth Cohort Study (START) will investigate the environmental and genetic basis of adiposity among 750 South Asian offspring recruited from highly divergent environments, namely, rural and urban India and urban Canada.

**Methods:**

Detailed information on health behaviors including diet and physical activity, and blood samples for metabolic parameters and DNA are collected from pregnant women of South Asian ancestry who are free of significant chronic disease. They also undergo a provocative test to diagnose impaired glucose tolerance and gestational diabetes. At delivery, cord blood and newborn anthropometric indices (i.e. birth weight, length, head circumference and skin fold thickness) are collected. The mother and growing offspring are followed prospectively and information on the growth trajectory, adiposity and health behaviors will be collected annually up to age 3 years. Our aim is to recruit a minimum of 750 mother-infant pairs equally divided between three divergent environments: rural India, urban India, and Canada.

**Summary:**

The START cohort will increase our understanding of the environmental and genetic determinants of adiposity and related metabolic abnormalities among South Asians living in India and Canada.

## Background

**Figure 1:**
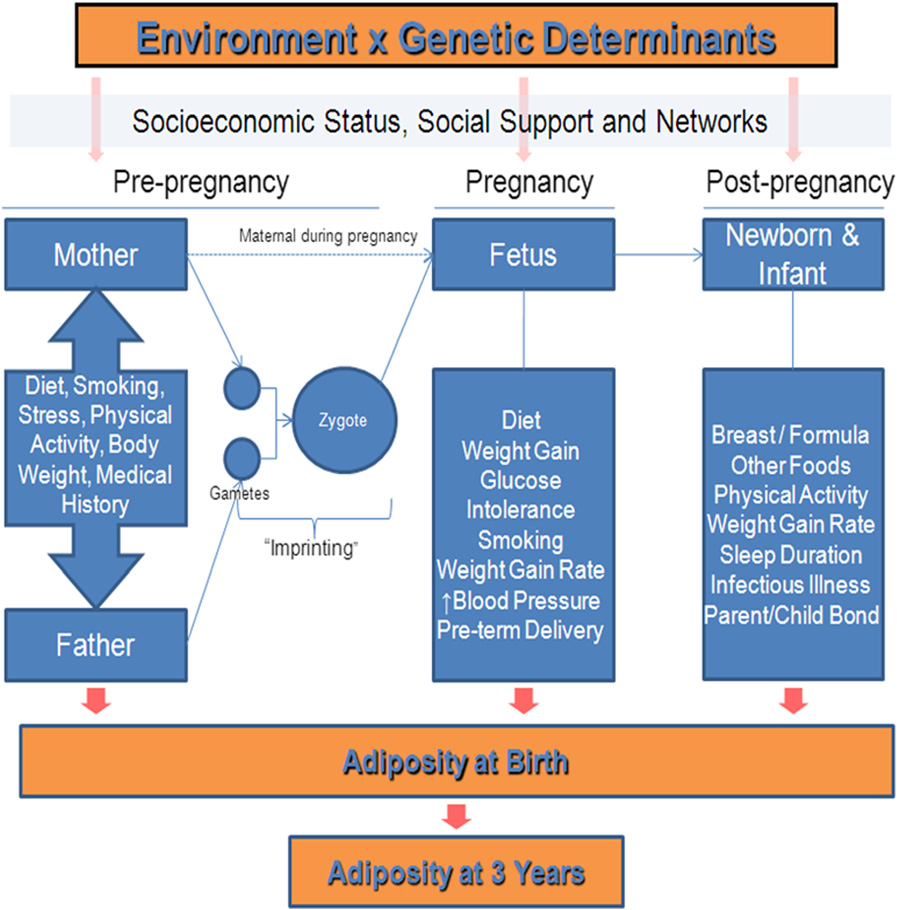
Hypotheses to be investigated in the START Cohort.

Globally, people of South Asian origin are at high risk of developing type 2 diabetes and cardiovascular disease (CVD) [1]. In India, the prevalence of type 2 diabetes is currently estimated to be 40 million, with an expected rise to 79 million by 2030 [2]. The propensity to develop type 2 diabetes is observed among South Asians living in urban environments in India and among the rapidly growing South Asian population living in Canada [3-6]. Previous studies have suggested that compared to white Caucasians, South Asian newborns have relatively lower birth weight and yet possess more adipose tissue [7]. This thin-fat phenotype at birth may lead to alterations in metabolic risk factors early in life and may track into adolescence and adulthood [8]. To better understand the development of adiposity among South Asians from India, we have initiated a transcontinental study of the environmental determinants of adiposity among 750 South Asian offspring recruited from highly divergent environments, namely, rural and urban India and urban Canada (Figure 1).

**Table 1:** Inclusion and Exclusion Criteria

**Inclusion Criteria**	**Exclusion Criteria**
Women Age: 18-40 years	Expected Multiple Births
South Asian Origin	Artificial/Assisted Conception of Fetus
Pregnant with Single Fetus	
	> 4 Live Births
	Surrogate Mothers
	Chronic Medical Conditions
	- Active Cancers
	- HIV
	- Hepatitis B or C
	- VDRL Positive
	- Rheumatic Heart Disease
	- Seizure Disorder
	- Living in Canada < 9 months
	- Father of the baby is not of South Asian origin

## Study rationale

### South Asians unique risk factor profile

People of South Asian origin are among the highest risk groups for the development of type 2 diabetes and CVD with excess adiposity as the apparent underlying cause [9]. Almost half of all deaths in South Asia are now attributable to non-communicable diseases which account for 47% of global burden of disease [10]. This propensity to develop type 2 diabetes is observed among South Asians living in urban environments inside India, and in those living outside of the country [11]. Studies of South Asian migrants to North America, the United Kingdom, and South Africa, indicate that South Asians have a much higher risk of dying from coronary artery disease (between 1·5 and 4·0 times) compared to other ethnic groups [12]. Further, South Asians have more adipose tissue and develop abnormal glucose, lipids, and blood pressure at a significantly lower body mass index (BMI) compared to white Caucasians [13]. For example, South Asians demonstrate increased plasma glucose, apolipoprotein B and systolic blood pressure and lower apolipoprotein A at lower BMI levels compared to white Caucasians who develop equivalent changes at higher BMI levels (i.e. above 30) [13,14]. The likely pathophysiology of South Asians’ heightened sensitivity to weight gain is their increased propensity to develop visceral abdominal fat and fatty infiltration of the liver associated with excess energy intake [14]. The accumulation of visceral and ectopic fat is associated with metabolic syndrome traits (i.e. increased glucose, abnormal lipids and elevated blood pressure) [15]. It is critical to understand the environmental and genetic determinants of these factors because collectively these risk factors account for over two-thirds of the population attributable risk of myocardial infarction [16].

### South Asians living in India

The risk factor profiles among South Asians living outside of India compared to those living within urban India are similar [17]. The most dramatic differences in risk factors are observed between urban and rural regions within India. A study by Kumar et al. among men aged 35-54 years showed that, hypertension was 11 times higher (38.2% vs 3.4%), type 2 diabetes was 21 times higher (6.2% vs 0.3%), the proportion of population with a BMI ≥25 was 12 times higher (34.1% vs 2.9%), and physical inactivity was 5 times higher (47.2% vs 8.6%) among the urban compared to rural dwellers [18]. Thus, the CV risk factors among South Asians within and outside of India are strongly correlated to weight gain which develops due to urban lifestyle.

In addition to energy intake and relative proportion of macronutrients consumed by mother, micronutrient deficiencies during pregnancy have been associated with multiple potential health consequences in the newborn [19]. Yajnik and colleagues reported that high folate intakes in vitamin B12-deficient mothers increases the risk of insulin resistance and type 2 diabetes in the offspring indicating that the defects in one-carbon metabolism might be important in the intra-uterine programming of adult disease [20]. Data generated from our cohort of pregnant women in urban Bangalore, India, showed that women in the lowest tertile of serum vitamin B12 concentration during each of the three trimesters of pregnancy had a significantly higher risk of delivering a growth restricted baby (OR, 5.98, 9.28 and 2.81 for trimesters 1-3, respectively), when compared to women in the highest tertile [21].

### South Asians living in Canada

There are more than 1.2 million people of South Asian origin living in Canada [4]. In the *Study of Health Assessment and Risk in Ethnic groups* (SHARE) [5] among a random selection of South Asian and European adults in Canada, the age-adjusted prevalence of type 2 diabetes and CVD were over two-fold higher in South Asians than in Europeans [5]. Furthermore, South Asians in Canada had higher fasting levels of glucose and insulin and were significantly more “insulin resistant”, as assessed by the homeostasis model assessment index [13]. Notably, this was the case, despite BMI and waist circumference being marginally greater in Europeans than in South Asians, consistent with observations by other investigators [6,22]. Comparisons of health behaviours of South Asians to white Caucasians in Canada indicate that although total energy consumption is similar, South Asians consume a greater amount of carbohydrate, mostly from foods with a high glycemic index including traditional breads (such as chapatti and roti) and rice [23]. In addition to unique dietary patterns among South Asians in Canada, they also appear to be relatively sedentary, as they report less work-related physical activity, time spent playing sports, and leisure time activities compared to Europeans [5]. These health behaviors may account in part for South Asians’ unique preponderance to develop abdominal obesity, and raise the possibility that the dietary and physical activity patterns may partially explain the unique South Asian risk factor profile.

### Fetal origins of adiposity among South Asians

There is evidence to support the concept that adiposity and related metabolic factors may be influenced by factors which “program” the developing fetus [24]. Data from birth cohort studies conducted in India compared to UK newborn data showed that South Asian mothers were younger and had a lower BMI (18 vs. 23) compared to the European mothers, and that South Asian babies were lighter (2.7 kg vs. 3.5 kg), shorter, yet had comparable subscapular skin fold thickness, suggesting for their weight they had relatively more adipose tissue [7,8]. These South Asian babies were also found to have increased adiposity, glucose, insulin and leptin concentrations in later life (at 6 to 8 years). However these observations have not been consistently reproduced by a subsequent urban-based birth cohort from Bangalore [25]. In this study, the mean birth weight of all urban and peri-urban newborns was 2.80+/−0.44 kg, and the skin-fold thickness among these babies were similar to those reported in a Western population with comparable birth weights [25]. To date there has been no detailed study of the anthropometric characteristics of South Asian newborns in Canada. Recently, Ray reported that the mean birth weight for 753 South Asian babies born between 2002-2007 at 39 weeks gestational age was 3.2 kg. While this was significantly lower than the mean birth weight of European origin babies in Canada (3.4 kg, P<0.001), it is substantially higher than the reported birth weights from the rural and urban birth cohorts of South Asians from India. (2.7 and 2.8 kg respectively) [26]. However these between center comparisons are indirect, and it is possible that differences in maternal characteristics, gestational age, and pregnancy factors (i.e. maternal nutrition, gestational diabetes, hypertension) may explain these differences. Thus the conflicting reports from urban and rural areas of India, (the reported studies are also separated temporally by about a decade), and sparse Canadian data in South Asian babies underscore the need for the creation of a birth cohort of South Asian mothers and offspring. The advantage of studying South Asians adults and their offspring in urban and rural areas of India and urban Canada is the heterogeneity of environment across regions (i.e. body weight, diet, micronutrient status, physical activity, social and cultural factors) which together with genetics, will enable us to understand the key early life determinants of childhood adiposity in this high risk group. This information will also help us to understand the role of fetal programming in the development of adiposity in South Asians in general - a high risk group for type 2 diabetes and CVD.

## Primary study objectives

1. To characterize the *in-utero* environment by assessing the influence of antenatal maternal, paternal factors (e.g. medical history, dietary intake, smoking exposure, and psychosocial stress), and pregnancy factors (e.g. abnormal gestational glucose tolerance, pregnancy-induced hypertension, preterm births, and newborn small and large-for-gestational age birth weight) across diverse environments on newborn’s body composition, birth weight and length.

2. To study the association between early feeding practices (i.e. breastfeeding, weaning and complimentary feeding pattern) and post-natal growth and adiposity at 1 and 3 years after birth across diverse environments.

3. To study the association between specific micronutrient status (vitamin B12, homocysteine and folate) and gestational weight gain with body composition and birth weight across diverse environments.

Secondary Objectives:

4. To determine the impact of the home environment, including family structure, socio-economic status, health behaviours (i.e. dietary intake and physical activity) maternal and paternal psychosocial stress factors, and parent-child interaction, on the development of adiposity of the growing offspring in the first three years.

5. To determine the association between birth weight and measures of adiposity at birth with morbidity events (respiratory infections, unspecified fever and diarrhoea) and its impact on anthropometric measures during the first year of life across diverse environments.

6. To validate measures of adiposity (i.e. triceps skinfold thickness) with accurate measures of body composition including deuterium dilution technique, bioelectrical impedance assessment (BIA) and DXA scanning.

The START prospective cohort study will also create a platform to enable a broad range of scientific questions beyond the primary and secondary objectives to be addressed including:

a) Comparisons of measures of body adiposity using skin-fold, bioimpedance, and deuterium dilution technique.

b) Determination of the relative contribution of selected candidate genes and epigenetic markers using DNA collected from cord blood at birth to adiposity at birth and adiposity accumulation in the growing offspring to 3 years

c) Characterizing the infant microbiome and investigating the interaction between the microbiome, diet, and adiposity.

## Methods and design

START has been granted ethical approval locally from the Research Ethics Board, Hamilton Health Sciences/McMaster Health Sciences (REB#: 10-640) and in India, Institutional Ethics Review Board Reference #: 114/2010). In both countries, pregnant mothers are recruited during their antenatal visits (1^st^ or 2^nd^ trimester) to their primary care practitioner or obstetrician. The study is described by the study personnel to the pregnant mothers and consent for participation is obtained. Information concerning medical and pregnancy history, health status, health behaviors, and socioeconomic status is obtained by questionnaires. Anthropometric measurements (height, weight, skinfold thickness), blood pressure, urine sample, and a fasting blood sample for glucose, insulin, micronutrients (i.e. vitamin B12, RBC folate, plasma homocysteine, methylmalonic acid MMA), lipids and a buffy coat for future DNA extraction will be collected, and processed using a standardized protocol at 24-28 weeks of gestation. Mothers who are not known to have diabetes will undergo a 75 oral glucose tolerance test between 24-28 weeks gestation. The results of an ultrasound performed between 18-24 weeks to assess for congenital anomalies and for precise determination of gestational age will be collected from each pregnant mother. At the time of delivery, details of the delivery, birth outcomes for the mother and baby will be collected, and a cord blood sample for DNA, glucose, insulin, lipids and additional aliquots for future analysis of adiponectin, and leptin will be taken from each baby. The placenta will be weighed, and where possible a biopsy of the placenta will be collected and stored in RNAlater for future analysis of RNA and methylation patterns. In addition, the infant’s anthropometry including birth weight, triceps and sub-scapular skin fold thickness, length, abdominal, head, and arm circumference will be measured by a trained research assistant.

### Inclusion criteria

Women between 18-40 years of age who are pregnant with a single fetus who are either from rural or urban India, or are of South Asian origin living in Canada (defined as maternal and paternal grandparents originating from the Indian subcontinent) are eligible.

### Exclusion criteria

Women with expected multiple births, who have had more than 4 live births previously, surrogate mothers, women who conceived the fetus using artificial methods including in-vitro fertilization or intrauterine insemination, women who suffer from severe chronic medical conditions including active cancer, severe infectious diseases including HIV, hepatitis B or C, or who are VDRL positive, women with significant heart disease (i.e. rheumatic heart disease), or a seizure disorder requiring daily medication will be excluded (Table 1).

## Communities included in start

The urban cohort in India is recruited from the St. John’s Medical College Hospital, a charitable 1,200 bed tertiary hospital serving a broad mix of urban middle class and urban poor in Bangalore city, India's third largest city (population of 6.5 million), Karnataka State. Because of its proximity to both residential areas that house low income and middle class families, it draws patients of diverse socioeconomic status. The obstetric department conducts around 2000 - 2500 deliveries per year.

The rural cohort is drawn from Snehalaya Hospital located in the village of Solur, Karnataka State. This rural mission charitable hospital provides nearly free health care to the rural population and the hospital conducts about 1,200 deliveries per year. The majority of the residents in Solur district are manual laborers involved in agrarian work. Their annual income is about 15,000 INR (equivalent to 266 Canadian dollars) with poor educational attainment (80% educated up to primary school).

### Canada

The cohort in urban Canada is recruited in the Peel Ontario Region where South Asians account for 18% of the population. Through referrals from primary care and specialist physicians, participants are approached and assessed at one of three hospitals - the Brampton Civic, Credit Valley and Trillium Hospitals. The Brampton Civic hospital is a high volume community hospital with 4,460 newborn deliveries/year, of which approximately 40% are to South Asian mothers. Credit Valley Hospital and Trillium Health Centre each report 4,695 and 4,130 birth respectively per year and approximately 20% are South Asian. Follow-up visits occur at the hospital where the mother delivered.

### Inter-country measurements

All questionnaires, physical measurement protocols, adiposity assessments, and blood collection protocols for key measures will be standardized between the three centres. The selection of measurement tools will be harmonized where possible, with some exceptions. For example, due to low acceptability of the deuterium dilution technique measurement of body fat by South Asian mothers in Canada this is not be performed in Canada. Second, some questions were only relevant to South Asians living in Canada including acculturation to a new country. Third, while individual questions to assess social and economic factors are similar between the cohorts, the scales differ as they were developed and validated in different countries. Specific measures, which will be obtained from participants in India and Canada, are provided in detail below.

## Stage 1: antenatal assessment

### Socio demographic information

Trained research assistants collect socio-demographic data through questionnaires on information on age, parity, family structure, and number of children in the house. Information of the medical and prior obstetric history is recorded. Socioeconomic factors including household income, education, and employment is also collected. The standard of living index in India will be calculated using Parsuraman’s standards [27]. This index was used in the National survey by Government of India and provides information on income, employment, and educational attainment and is applicable to rural population. In Canada, information on annual household income, employment, educational and marital status will be obtained through the social disadvantage index developed in the SHARE study [28].

### Maternal anthropometry

Detailed anthropometric measurements of all pregnant mothers will occur during the second trimester. A digital scale (Soehnle, Germany) is used to record the weights of the subjects to the nearest 100 g. Height is measured using a stadiometer to the nearest 1 cm and mid upper arm circumference to the nearest 0.1 cm using a plastic measuring tape. Maternal BMI is calculated using pre-pregnancy weight and height (kg/m^2^). Maternal weight gain will be determined comparing the maternal weight at the time of delivery to the pre-pregnancy weight. At the initial visit skinfold thickness (biceps, triceps and subscapular) is measured to the nearest 0.2 mm, using skinfold calipers (Holtain, Crymmych, UK), for the prediction of body density from which, body fat and fat free mass (FFM) is estimated using prediction equations [29]. Mid-upper arm circumference (MUAC) is measured with a plastic tape. Midarm muscle area (MAMA) will be calculated using the following equation:

$$\text{MAMA}=\frac{{\left(\text{MUAC}\;-\;3.14159\;*\;\text{TSF}\right)}^{2}}{4\;*\;3.14159}$$

### Maternal glucose status

The diagnosis of maternal glycemic status is made using standardized methods at similar time point in pregnancy across the 3 cohorts. All non-diabetic mothers undergo the 75 oral glucose tolerance test between 24-28 weeks of gestational age. This test is chosen to avoid the high false negative rate using the 50 gram Glucose Challenge Test among South Asians. Blood samples are stored for future measurement of maternal insulin sensitivity by analysis of stored samples for fasting insulin at the time of the OGTT [30].

### Dietary assessment

Maternal diets are assessed in pregnancy and in follow-up (1 and 3 years after delivery) using validated food frequency questionnaires (FFQ’s). There are differences in the feasibility and administration of these measures between India and Canada, but common time points of measurement for dietary assessments will include pregnancy, early post-partum, and after weaning to assess maternal diet.

### India FFQ

This FFQ focuses on the diet intake for the preceding 3 months and will be administered once in each trimester (at recruitment, 12±1 week of gestation i.e. 1st trimester, 24±1 week of gestation i.e. 2nd trimester and again at 34±1 week of gestation i.e. 3rd trimester. This questionnaire has been adapted from that developed for the urban middle class residing in South India [31], and has a food list of 108 items, derived from a food database developed over a period of many years from studies at the Division of Nutrition, St John’s Medical College, and four frequency categories (daily, weekly, monthly and yearly). Nutrient compositions of the food item are calculated using standard food conversion tables for the ingredients [32]. Wherever available, Indian data are used. However, for some nutrients, for which Indian data is not available, USDA data in the public domain are used [22]. The program computes nutrient scores by multiplying the relative frequency of consumption of each food item by its nutrient content of the standard portion size. Nutrient information is obtained on 27 macro- and micro-nutrients. Similar FFQs have been validated against 24-h food recalls (mean of three records per trimester) at each trimester of pregnancy. Information on the use of routine antenatal dietary supplements during each trimester of pregnancy will also be collected.

### Canadian South Asian FFQ

The FFQ used in the Canadian cohort was developed and validated for use among South Asians living in 3 regions in Canada, Toronto, Hamilton and Edmonton [33]. The FFQ was validated against 7 to 14 day food records and the energy adjusted, deattenuated correlation coefficients were 0.45-0.57 for protein, 0.17-0.62 for total fats, 0.31-0.60 for carbohydrates, and 0.63-0.70 for total fiber [33]. On the FFQ, participants report how often, on average, they consumed selected foods in the previous year. We calculate nutrient intakes by multiplying the average nutrient content of a particular food portion by the number of times it was consumed. Nutrient content (macro and micronutrients) will be determined by linking the FFQ to the South Asian specific nutrient database in which the food composition data for each FFQ item of the South Asian FFQ is contained [33]. This nutrient composition database was created in the Food Processor nutrient analysis software (version 6.11, 1996, ESHA, Salem, OR), which incorporated the 1991 Canadian Nutrient File and US Department of Agriculture databases. The nutrient composition of common food items (raw fruits, vegetable) were taken from the Food Processor program. However, the nutrient composition for unique South Asian recipes was created by first creating a prototype recipe for common items (e.g. chicken curry) based on the different recipes of the same item reported in individual diet records. The nutrient composition of this prototype recipe was then created in the same Food Processor program and is used to determine nutrient composition in the FFQ nutrient database [33].

### Physical activity

Maternal physical activity is assessed to capture information on occupational activity outside the house, discretionary exercise, household chores, and sedentary activities. In India, a previously validated physical activity questionnaire is administered to assess the activity level in the pregnant women during each trimester [34]. Information is collected for activities in 5 domains - occupational activity outside the house, discretionary exercise, household chores, sedentary activities, hobbies and sleep. Physical activity data is expressed as the duration (minutes/day). In Canada, a similar activity assessment tool is used, and includes the same domains of activity outlined above. Specifically, activity level on average is assessed both qualitatively, through classification as “sedentary”, “mild”, “moderate”, or “strenuous”, and quantitatively as minutes per day spent engaging in sedentary behavior, strenuous exercise, physical labour, and household chores. The common metric to compare activity levels between regions in START is minutes of activity per day which can be subdivided into type of activity, i.e. work related activity, household chores, or leisure time activity. In addition sedentary behaviours such as screen time per day using the computer, television or playing video games are collected in India and Canada. This information is collected from the mother during the second trimester of pregnancy, 12, 24, and 36 months after delivery.

### Assessment of psychosocial stress

Information on psychosocial stress is collected at baseline and includes questions about maternal depression and social support. Subjective socioeconomic status is measured using the MacArthur Scale of Subjective Social Status [35]. This scale asks mothers to place their household on a ladder relative to their community with higher rungs representing higher status. This scale will be administered in Canada at the 1 year follow-up visit.

i. **Maternal Depression**: Depression in the mother is assessed by the Kessler-10 scale for screening of depression (K-10). Developed for use in the US National Health Interview Survey, the K-10 is a short questionnaire developed to screen for depression by determining a composite score based on participant’s responses [36]. The K-10 is a 10-item scale with five response categories ranked on a 5-point scale with the score being the total sum of scores (range 0-50). In an Indian study comparing five screening questionnaires to detect common mental disorders, the K-10 showed a high degree of correlation with other commonly used screening questionnaires in the detection of depression [37]. In addition, the K-10 was found to be a valid measure of depression in the postnatal period in a developing country setting [38]. We have recently used the K-10 as a measure of both antenatal psychological distress and it compared well with other scales routinely used to measure depression during pregnancy [39]. The K-10 is administered by trained personnel during pregnancy and during the postnatal period at 6 and 12 months in India and at 3, 6 months and 12 months in Canada. An algorithm has been developed to ensure appropriate intervention and follow-up for any mothers in Canada who are identified as depressed or suicidal. (http://southasianbirthcohort.com/Research/Protocol).

ii. **Social Support**: Adequacy of social support to the mother is measured in both countries using a questionnaire which was developed at St. John’s Research Institute to evaluate a broad range of social support (i.e. emotional, instrumental, informational, and appraisal) [40]. The social support questionnaire is administered in the second trimester of pregnancy and then at 12 months post-partum. The assessment of social support has some common questions and some different domains given the diversity in cultural context and differences in home environments we anticipate. In both countries we will collect information on numbers of family members living in the home and the ease by which relatives can be called upon during times of need.

iii. **Acculturation and Intimate Partner Violence (Canada only):** As acculturation to a new environment is a unique component to the South Asians who reside in Canada, only participants in Canada will acculturation stress be measured. The measurement tool included the Vancouver Inventory Assessment (VIA) which is a validated questionnaire that has been used among people of South Asian origin living in the United States. This questionnaire will be self-administered at the one year follow-up visit [41]. In addition mothers will be asked to complete the HARK questionnaire [42]. This questionnaire consists of 4 questions that screen for physical, sexual and emotional violence in the past year. The HARK questionnaire will be administered by trained personnel at the 1 year follow-up visit in Canada. Any women that are identified to be at risk of intimate partner violence will be offered appropriate support and referral.

### Fetal growth characteristics

An ultrasound examination will be carried out in all fetuses during the first or second trimester of pregnancy. The fetal sonograms will be used for both establishing gestational age and assessing fetal growth characteristics. Fetal growth measurements include head circumference and abdominal circumference in the second trimester measured to the nearest millimeter by using standardized ultrasound procedures.

## Stage 2: delivery and newborn data collection

Anthropometric measurements of the child will be collected at birth, 6 months and 12 months of age in all cohorts. Infants will be weighed to the nearest 10 g on a digital baby weighing scale; length will be measured on an infantometer. Ponderal index of the child will be computed using length and weight by the formula- mass/height. Head, chest and mid upper arm circumference of the baby will be measured to the nearest 0.1 cm using a plastic measuring tape. Skinfold measurement will be measured to the nearest 0.2 mm, using skinfold calipers (Holtain, Crymmych, UK) for prediction of body composition. All measures will be done by trained personnel, and inter-observer reliability testing will be conducted.

### Specific measures include

Crown-heel length which will be measured using a regularly maintained and calibrated length board until 18 months of age and height will be measured using a Harpenden stadiometer in those > 18 months of age. Weight will be measured with an electronic scale with the subject in a single layer of outer clothing. BMI will be calculated (weight/height^2^) and percentiles for weight, height and BMI for age and sex obtained using the WHO reference growth charts [43]. The international standard for overweight and obesity based on BMI will be used.

### Skinfold thickness

Skinfold thickness will be measured at the antenatal visit for mothers and at birth and subsequent 1, 2, and 3 year visits for infants. Skinfold calipers will be used to make this measurement to the nearest 0.2 mm by trained study personnel. Triceps skin fold thickness is measured at the midline of the posterior aspect of the right arm, over the triceps muscle, at the mid-point of the arm identified with elbow flexed 90 degrees. Subscapular skin fold thickness is measured with subject standing tall, arms comfortably hanging at the side. The skinfold should be slightly inferior to the angle of the scapula.

### Bioelectrical impedance analysis (BIA)

Body fat of the offspring at 3 years of age will be assessed using Quantum II BIA analyzer (RJL Systems). By applying alternating current to the body tissues (not felt by the individual), BIA measures electrical impedance of tissues, which is used in regression equations to approximate total body water. Using total body water then, the body fat and fat free mass are calculated. The BIA is non-invasive, portable and requires only a few minutes.

### Deuterium dilution technique in rural and urban babies in India

In a subset of the India urban cohort, the body fat of the babies will be measured from the total body water content of the subjects, measured by ^2^H dilution [44]. Details of this measurement technique are found on the START website (http://southasianbirthcohort.com/Research/Protocol). In India the body composition using deuterium dilution technique will be assessed at 1 and 3 years in a subset of urban cohort [45].

### Biological specimen collection

At birth**,** the placenta will be weighed (untrimmed placental weight) and a full depth section of 1.5 x 1.5 cm will collected from central region between the core and periphery. This sample will be stored in RNAlater (in a refrigerator for a 10-12 hour period prior to freezing) and will be frozen in liquid nitrogen this sample will allow for extraction of DNA and RNA. **Cord blood** will be collected from the umbilical vein after the cord has been cut and before placenta delivery. A total of 18.5 mL of blood is collected to obtain, plasma, serum, buffy coat (for DNA) and PaxGene (for RNA) samples. All sites will follow a central protocol for sample collection, processing, and aliquoting. In Canada samples from the three recruiting hospitals will be periodically shipped to the central storage at the Clinical Trials Clinical Research Laboratory, Hamilton Health Sciences, where they will be stored in liquid nitrogen for later measurement of lipids, insulin, and glucose and for genetic testing. Additional serum samples will be stored for future analyses. In India all samples will be processed and stored in liquid nitrogen at the St. John Research Institute.

## Stage 3: data collected after neonatal stage

**Figure 2:**
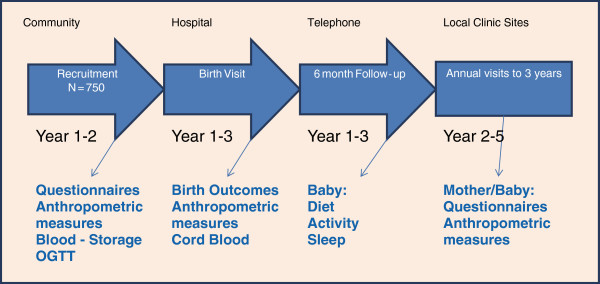
START Enrolment and Follow-up.

Given the differences in health care systems and feasibility of recruitment between India and Canada, the timing of follow-up assessments will vary somewhat, although both countries will collect the baby’s 2 week body weight, and monthly weight and length which will be collected by telephone interviews or by face to face examinations. In addition both countries will conduct annual assessments of the baby and mother dyad until the baby is 3 years of age. (Figure 2) The immunization of the child will be recorded at each follow up visit. A detailed schedule outlining study visits for all three cohorts is found in Table 2.

**Table 2:** Timing and Type of measurement in START Canada and India

**Measures**	**Antenatal Visit (m)**			**Birth (m, b)**	**First Year (m, b)**			**12 Month (m, b)**	**24 Month (m, b)**	**36 Month (m, f, b)**
	**T1**	**T2**	**T3**		**3 mth**	**6 mth**	**9 mth**			
**Demographics**										
Age	I	C								
Expected Delivery Date	I	C								
Medical and Obstetric History										
Gravida	I	C		C (m)						
Current gestational diabetes	I	C								
Current increased blood pressure	I	C								
Increased cholesterol	I	C								
Other Major Medical conditions Checklist	I	C								
Family History	I	C								
Medications Used	I	C								
Past Pregnancy History										
Live Births	I	C								
Still Births	I	C								
Abortions (therapeutic and spontaneous)	I	C								
Past Gestational diabetes	I	C								
Pre-Eclampsia	I	I/C								
Multivitamin Use	I	I/C								
**Social Determinants**										
Self-reported Ethnicity - for self, parents and grandparents	I									
Mother tongue	I	C								
Religious denomination	I	C								
Years in Canada		C								
Place Immigrated from	I	C								
Place of Birth	I	C								
Annual Household Income	I	C								
Occupation	I	C								
Marital Status	I	C								
Education	I	C								
Social Support	I	C						I/C (m)		
Social Status-McCarthur								I/C		
Acculturation								C (m)		
Alcohol and tobacco use		C								
Maternal Depression		I/C				(m)		I/C (m)		
Intimate Partner Violence								C(m)		
Home Environment and Parenting								I/C (m)		
Infant Temperament								I/C	I/C	I/C
Sleep Patterns					C	C		I/C		
**Diet**										
FFQ		I (mf)/C (m)		I (b)				I/C (m)		I/C (m)
24 Hour recall	I (m)	I	I(m)		I	I (m)	I	I/C (m)		I/C (mother for baby)
Infant Feeding questions				I	I/C	I/C		I/C		I/C
Physical Activity	I	I/C	I					I/C (m)	C (m)	I/C (m)
**Anthropometric measures**										
Blood Pressure	I	I/C	I	I/C (b)				I/C	I/C	I/C
Height/Length	I	I/C	I	I/C (b)	I/C (b)		I	I/C		I/C
Weight	I	I/C	I	I/C (b)	I/C (b)	I/C (b)^1^	I	I/C	I/C	I/C
Waist and Hip Circumference	I	I/C	I					I/C	I/C	I/C
Skin Folds	I	I/C	I	I/C (b)	I		I	I/C (b)	I/C (b)	I/C (b, m)
Head circumference				I/C (b)	I/C	I/C	I	I/C (b)		I/C (b)
Chest, mid upper arm Circumference				I/C (b)	I	I	I	I/C (b)		I/C (b)
Fetal Ultrasound		I/C (m)	I/C (m)	I (b)				I (b)	I (b)	I (b)
DLW								I	I	
BIA										I/C (b)
Blood Specimens										
Hemoglobin	I (m)	C (m)		I/C (b)				I/C (m)		I/C (m, f)
Fasting Glucose		I/C (m)	I	I/C (b)				I/C (b)	I/C (b)	I/C (b)
75 g OGTT		I/C (m)								
Insulin		I/C (m)	I	I/C (b)				I/C (b)	I/C (b)	I/C (b)
Lipids		I/C (m)	I	I/C (b)				I/C		I/C
ALT		I/C (m)								
Micronutrients (B12 Folate)		I/C	I							
DNA/long term Storage (urine sample)				I/C (m)						
Birth Visit				I/C (m)						
Type of Delivery				I/C (m)						
Pregnancy weight gain				I/C (m)						
Duration of Labour				I/C (m)						
Low Birth Weight				I/C (b)						
Premature Delivery				I/C (b)						
Premature Labour				I/C (m)						
Blood Loss				I/C (m)						
Epidural				I/C (b)						
Birth Weight				I/C (b)						
APGAR scores (1 min & 5 min)				I/C (b)						
Vitamin K Given				I/C (b)						
Adverse outcomes				I/C						
Placenta				I/C						
Cord Blood				I/C (b)						

Legend: *I* India, *C* Canada, *b* baby, *m* mother.

### Breastfeeding practices and infant diet recall

Information on child-care and feeding practices will be collected using standardized and validated questionnaires by trained research assistants during face-to-face or telephone interviews with the mother or other caregiver of each participating infant/child. Information on initiation of breastfeeding, exclusivity of breastfeeding, duration of breast feeding, prelacteals and introduction of complementary foods will be collected. In India at 1 month, 6 months, and yearly until 3 years of age, and in Canada this will be collected at 3 months, 6 months and 1 year and thereafter annually until three years of age. An Infant feeding form will be used for data collection. The form is a closed ended questionnaire with information about breastfeeding, use of formula and type of formula, the introduction of solid foods, and the type and amount of solid foods consumed. At the 2 and 3 year visit, the mother will complete a 24 hour recall for the child. Information on supplements and home remedies for acute illnesses will also be recorded. Information on the child morbidity, medications and hospitalizations will also be noted at each follow-up assessment.

### Sleep patterns

Information on sleep patterns will be collected using the Brief Infant Sleep Questionnaire (BISQ) [46] by trained research assistants during face-to-face or telephone interviews at 3, 6, and 12 months. The BISQ is considered a reliable and valid tool for the measurement of sleep problems in infants. It has been used successfully in studies with both Canadian and South Asian infants and toddlers [46].

### Home environment, parent behavior, and child temperament

We will capture aspects of home environment and parent-child interaction, bonding, and child temperament. In India, the Bradley questionnaire will be used to capture information on parenting behavior under 6 domains (responsively, acceptance, organization, learning materials, parental involvement and variety of stimulation at home) [47]. This questionnaire will be administered by trained study personnel to participating families at 1 year after delivery. Canada will use a self administered Home Screening Questionnaire at the 1 year visit. These two measurement instruments collect information on common domains of parental discipline, playtime, pets, parent child interactions, bonding, alternative care-givers, and home environment stimulation. Child temperament will be assessed in India and Canada at the annual visit using the Carey Temperment scales (CTS) appropriate for the corresponding age of the child [48].

### Standardization of measurements across labs

Common analytical instruments are present in both research institutions in Canada and India and standardized assays for hematological and biochemical analysis will be undertaken. After 100 mothers and babies have been recruited from all 3 centres, an evaluation of the data quality will be made before proceeding to complete the full cohort recruitment. As noted above, common analytical instruments are present in both research institutions in Canada and India and standardized assays for hematological and biochemical analysis will be undertaken. Both laboratories will test the same set of prepared pools on each analytical run as part of the quality control system, to assess within laboratory precision and differences between the two laboratories. We have developed a stringent data management system for use in our ongoing studies.

### Statistical power

We estimate that 250 babies and mothers from each centre will provide substantial power to address the main objectives (750 South Asian babies in total), including identification of between region differences in maternal and offspring characteristics with high precision. The triceps skin fold thickness at birth will be the primary outcome measure across the three cohorts (rural and urban Indian and Canada). The sample size estimation is based on triceps skin fold measure. In an urban birth cohort study conducted in Bangalore the mean triceps skin fold was 3.8 mm (SD: 0.89) [25]. In a rural cohort from India, the mean triceps skin fold measure was 4.2 mm (SD: 0.74) [25]. Data on triceps skin fold measurement from babies of South Asian ancestry from Canada are not available. In order to detect a 0.5 mm difference in tricep skin fold measures between the three groups a sample size of 63 in each arm would be sufficient to examine the primary objective of the study with 80% power and at 1% level of significance. However as we are comparing three arms (urban and rural Indian and urban Canada) we must account for multiple comparisons using the Bonferroni correction. Considering this, and a 20% attrition rate the sample size to detect a 0.5 mm difference is 75 children in each arm. Furthermore inclusion of 250 babies per group will provide high power to detect a difference in skinfold thickness as small as 0.20 mm. In addition, we have high power to detect differences in other outcomes including birth weight. For example, using the estimated birth weight among South Asian babies in India, with 250 urban vs. 250 rural babies using a mean birth weight of 3.2 (0.65) kg we will have > 80% power to detect as small as a 0.22 kg difference between the cohorts.

### Statistical analysis

Descriptive statistics characterizing between region differences in maternal and newborn characteristics will be generated. Continuous variables will be reported using mean and standard deviation (SD) for the normally distributed variables otherwise median and inter-quartile range will be reported. Categorical variables will be reported using number and percentages. Normality of the variables will be examined using standard test with skewness and appropriate transformations applied if required. To determine if statistically significant differences exist between three groups, chi-square tests will be used and the specific comparison of region 1 vs region 2, region 1 vs region 3 and region 2 vs region 3 will be examined using the standardized residuals obtained for categorical variables and analysis of variance for continuous variables. Post hoc comparisons will be used to compare differences between the three groups, and correction of the P value to declare significance will be made. Linear regression will be used to assess the relation between the gestational weight gain and micronutrient status with the birth weight and adiposity at birth. Multivariate regression analysis will be used to find the determinants of adiposity at birth after adjusting for maternal factors and pregnancy factors. Changes in adiposity measurements over time will be compared by groups adjusting for confounders using linear mixed effects regression models. All analysis will be considered statistically significant at 5% level.

### Implications of this study

START is an transcontinental study designed to understand the early origin influences on the development of child adiposity among South Asians. By studying South Asians who are exposed to diverse environments we envisage these contrasts will yield etiologic clues to the South Asians predilection to develop excess adiposity, early age insulin resistance, and type 2 diabetes. First we seek to confirm or refute the “thin-fat” phenotype described by Yajnik [7]. Second, we will attempt to identify the key influences on newborn adiposity and birthweight by carefully measuring mothers dietary intake, physical activity, and glucose tolerance. Third, we will carefully characterize the home environment of the pregnant mother and her growing offspring to identify influences that are associated with maternal diet and activity and mental health. Fourth, once we have accrued ample sample size we will also be poised to study the influence of maternal genetics, fetal genetics, and their interactions with each other, and with environmental factors including postnatal feeding (type and amount) on adiposity, and weight at birth and in the first 3 years of life.

### Summary

There is an urgent need to understand maternal and child factors that underlie the early development of adiposity as the prevalence of childhood obesity is increasing especially among urban Indian children. This research proposal aims to prospectively follow South Asian mother-child dyads from both rural and urban settings in India and in Canada is novel by design and will provide unique and important information linked to early development of adiposity. This information is critical to enable development of prevention strategies for the emerging epidemic of childhood obesity and adult onset type 2 diabetes in India, and among people of South Asian origin who live in Canada.

## Competing interest

The authors have declared that they have no competing interests. START has not received funding/assistance from a commercial organization.

## Authors' contributions

SA, KS, MG, KT, AV, KM, AK conceived in the study design and participated in the writing of the manuscript. All authors read and approved the final manuscript. **India Principal Investigator include** Dr. K Srinivasan (PI) from St John’s Research Institute and St. John’s Medical College. **Canadian Principal Investigators include: Co-PI’s:** Dr. Sonia Anand, Professor of Medicine and Epidemiology from McMaster University in Hamilton, Ontario, and Dr. Milan Gupta, Associate Clinical Professor of Medicine McMaster University who is a staff cardiologist at the Brampton Civic Hospital and Medical Co-Director of the Canadian Cardiovascular Research Network. **Co-Investigators from St. John’s include:,** Dr. Anura Kurpad (Nutrition), Dr. TS Sridhar (Molecular Medicine), Dr. Jyothi S Prabhu, (Molecular Medicine), Dr. Swarna Rekha, (Pediatrics), Dr. Anil Vasudevan (Pediatrics & Division of Pediatric Nephrology), Dr. Annamma Thomas, (Obstetrics and Gynecology), Dr. Tinku Sarah Thomas (Biostatistics), Dr. Sr. Gladys Menezes, Director, Snehalaya Medical Relief Centre, Solur. **Co-Investigators from McMaster include:** Dr. Koon Teo (Medicine, Epidemiology, PI of FAMILY Birth Cohort study), Dr. Sarah McDonald (Obstetrics and Gynecology), Dr. Katherine Morrison (Pediatrics), Dr. Guillaume Pare (Genetics), Dr. Andrew Mente (nutritional epidemiology), Dr. Joseph Beyene (Biostatistics), Dr. James Dunn (Social determinants), Dr. Gita Wahi (Pediatrics), Dr. Rebecca Anglin (Psychiatry). **Collaborators:** Dr. Joel Ray (University of Toronto Maternal-Fetal Medicine), Dr. Vidiya Persad Maternal-Fetal Medicine Specialist at Brampton Civic Hospital, Dr. Jane Irvine (York University, psychosocial determinants), Dr. Michaela Hynie (York University, acculturation and social networks), Dr. Baiju Shah (University of Toronto: health services research), Dr. Ravi Retanakaran (University of Toronto: endocrinology). Dr. Carole Wade (Credit Valley Hospital site investigator), Dr. Peter Scheuffleur (Trillium Hospital site investigator), Dr. David Mowat, Medical Officer of Health for the Peel Region, Dr. Padmaja Subbarao co-PI of the CHILD birth Cohort study.

## Pre-publication history

The pre-publication history for this paper can be accessed here:

http://www.biomedcentral.com/1471-2458/13/79/prepub
